# Signal Quality Investigation of a New Wearable Frontal Lobe EEG Device

**DOI:** 10.3390/s22051898

**Published:** 2022-02-28

**Authors:** Zhilin Gao, Xingran Cui, Wang Wan, Zeguang Qin, Zhongze Gu

**Affiliations:** 1 State Key Laboratory of Bioelectronics, School of Biological Science and Medical Engineering, Southeast University, Nanjing 210096, China; 230189165@seu.edu.cn (Z.G.); wanwang@seu.edu.cn (W.W.); gu@seu.edu.cn (Z.G.); 2 Key Laboratory of Child Development and Learning Science, Ministry of Education, School of Biological Science and Medical Engineering, Southeast University, Nanjing 210096, China; qinzg@seu.edu.cn

**Keywords:** wearable, EEG, signal quality, rest, ERP, attention

## Abstract

The demand for non-laboratory and long-term EEG acquisition in scientific and clinical applications has put forward new requirements for wearable EEG devices. In this paper, a new wearable frontal EEG device called Mindeep was proposed. A signal quality study was then conducted, which included simulated signal tests and signal quality comparison experiments. Simulated signals with different frequencies and amplitudes were used to test the stability of Mindeep’s circuit, and the high correlation coefficients (>0.9) proved that Mindeep has a stable and reliable hardware circuit. The signal quality comparison experiment, between Mindeep and the gold standard device, Neuroscan, included three tasks: (1) resting; (2) auditory oddball; and (3) attention. In the resting state, the average normalized cross-correlation coefficients between EEG signals recorded by the two devices was around 0.72 ± 0.02, Berger effect was observed (*p* < 0.01), and the comparison results in the time and frequency domain illustrated the ability of Mindeep to record high-quality EEG signals. The significant differences between high tone and low tone in auditory event-related potential collected by Mindeep was observed in N2 and P2. The attention recognition accuracy of Mindeep achieved 71.12% and 74.76% based on EEG features and the XGBoost model in the two attention tasks, respectively, which were higher than that of Neuroscan (70.19% and 72.80%). The results validated the performance of Mindeep as a prefrontal EEG recording device, which has a wide range of potential applications in audiology, cognitive neuroscience, and daily requirements.

## 1. Introduction

Advances in wearable EEG monitoring technology introduce new opportunities for EEG collection outside the laboratory and long-term monitoring in research and clinical areas [1]. Any newly proposed wearable device requires rigorous verification, especially for clinical applications.

According to the wearable EEG research in the past 10 years, the application of wearable EEG mainly focuses on emotion, fatigue, sleep, rehabilitation training, and disease detection (e.g., epilepsy, depression and stress, etc.) [1]. The accuracy of the portable EEG device B-Alert x10 (Advanced Brain Monitoring, ABM) [2] and in-ear EEG in emotional arousal have been shown to be 75.00% and 72.89%, and 71.21% and 71.07% in valence, respectively [3,4]. Hwang [5] et al. used a wearable EEG to measure the emotional state of workers during construction tasks, and the results proved that wearable EEG features were correlated with cortisol and can be used to reflect the emotional state. In 2011, Looney [6] et al. proposed an ear-EEG device. Then, in the five-level sleep stage prediction experiment, the consistency between ear-EEG and the gold standard polysomnography device reached 74.1%, proving that the wearable EEG device can be used for sleep monitoring [7,8]. New wearable EEG devices, including Epitel Epilog [9], e-Glass [10], behind-the-ear EEG [11], Emotiv [12], and HealthSOS [13], have been shown to be useful for daily long-term monitoring of neurological diseases, such as epilepsy and stroke. Wearable EEG devices have the advantages of low power consumption, are light weight and portable, they are easy to operate, are low in price, and are more suitable for daily use and long-term monitoring in non-laboratory environments than traditional EEG devices.

There are diversified wearable EEG devices on the market, which can be divided into two categories: (1) Multichannel portable devices that require long battery life, and scalp-EEG of the whole brain result in larger and heavier equipment. As an example, B-Alert is a kind of portable device that is not suitable for daily wear. (2) Wearable EEG monitors that are more beautiful, light, and portable, and can be used in daily life, such as Emotiv, Muse, and Mindwave [2,14]. With its complete and mature hardware design and software support, Emotiv is currently the most widely used wearable EEG monitor in scientific research and business [15]. These wearable devices have good performances in areas including medical [16], sleep [17], emotions [18], depression [19], and fatigue [20]. Although several wearable EEG devices have appeared on the market, there were still some limits among them, such as low sampling rate, the absence of raw EEG data and no impedance detection (a detailed description and comparison will be shown in the Discussion section). Therefore, a new wearable prefrontal EEG monitoring equipment is proposed in this paper.

Before any wearable EEG device comes on the market and is widely used by the public, a rigorous signal quality evaluation experiment must be carried out to prove that the EEG data collected by the new device can be reliably used in research or daily life. The difference of power spectral density (PSD) in resting state EEG between eyes closed (EC) and eyes open (EO) is one of the most widely used evaluation methods for wearable EEG devices. The signal quality of the device in the frequency domain was proven reliable when the alpha (8–13 Hz) band power of EC was shown to be higher than EO [14,21,22,23]. The signal quality assessment in the time domain was verified through the event-related potential (ERP) experiments, including visual and auditory ERP [14,22,23,24,25,26,27]. Grummett [14] et al. compared Emotiv EPOC, B-Alert, g.Sahara, and g.HIamp. They found that the 16 and 23 Hz visual steady-state responses from the g.Sahara were statistically smaller than from the g.HIamp, B-Alert and Emotiv. The mismatch negativity (MMN) and P300 could not be obtained from Emotiv in the auditory ERP task. Kappel [22] et al. developed and evaluated a dry-contact ear-EEG. The signal-to-noise ratio (SNR) of the first harmonic responses of the ear–scalp were similar to the scalp–scalp electrode in auditory steady-state responses and steady-state visual evoked potentials task. The ear-EEG had the well-known P1-N1-P2 waveform in the auditory ERP task, and the MMN response was statistically significant for continuous intervals at the time of the most prominent peaks for both ear-EEG and scalp-EEG. In this study, a simulated signal test task and a comprehensive signal quality assessment of the proposed wearable device were completed.

Brain activity signals can be captured and studied by EEG signal processing methods and machine learning algorithms. SVM, decision trees, random forest (RF), naive bayes, K-nearest neighbors, and ensemble learning are commonly used machine learning models for supervised learning [28,29,30]. Hussain [28] et al. distinguish stroke patients from healthy people using delta, theta, and alpha activities and the C5.0 model, and achieved 78% accuracy for the resting state and 89% accuracy in the functional motor walking condition. EEG-based feature analysis combined with machine learning is a commonly used research method in emotion recognition [31,32,33]. Researchers in computer-aided EEG diagnosis are studying the different combinations of feature extraction methods and machine learning models as the diagnostic aid for disorders such as depression and epilepsy [34,35]. Aci [36] et al. used EEG features and classification models to distinguish three states of concentration, inattentiveness, and drowsiness. The SVM model performed the best, with an average accuracy rate of 91.72%. In our previous study [37], attention was divided into three levels: high, medium, and low, according to performance on the sustained attention task. Comparing the three classification results of SVM, RF, and XGBoost model, the XGBoost model showed the best performance with an accuracy of 80.42%. In this paper, the EEG features of high attention and low attention state were extracted, and the XGBoost model was used to distinguish high and low attention.

In this study, a new wearable frontal EEG device called Mindeep was proposed and the signal quality of Mindeep was evaluated to prove that it can be used in scientific and clinical research. The reliability of Mindeep’s hardware circuit was assessed by the simulated signal test experiment. Then, Mindeep was compared with the gold standard EEG monitoring equipment, Neuroscan, to verify that Mindeep can reproduce Neuroscan’s results in resting, auditory, and attention tasks. Compared with previous wearable EEG devices, the contributions of this study are as follows:

(1) Accurate transmission: Instead of traditional Bluetooth transmission, EEG signals with a high sampling rate and resolution recorded by Mindeep were transmitted to a computer at high transmission speed and accuracy via WIFI. WIFI can also be used to realize the synchronization of multiple devices in the local area network.
(2) Impedance detection: Mindeep supports the impedance detection function to monitor the skin-electrode impedance. This function improves the rigor of wearable EEG device during acquisition.
(3) Support both wet and dry electrodes: Mindeep supports both wet and dry electrodes to meet the needs of different application scenarios.
(4) Signal quality assessment solution: This study proposed a relatively complete signal quality assessment solution for different kinds of wearable EEG devices.
(5) Application case of wearable EEG devices: This study provided an application case in attention monitoring, which proved that wearable devices can be used in different applications to collect EEG in non-laboratory scenarios or long-term monitoring.

## 2. Materials

### 2.1. Device

**Figure 1:**
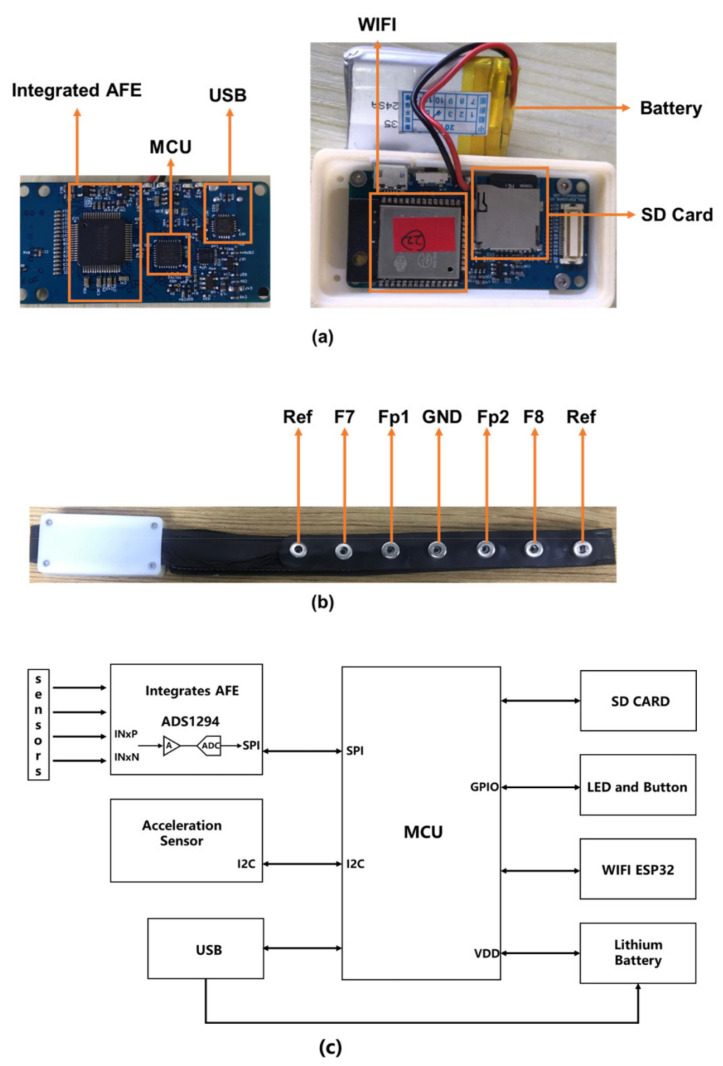
Wearable frontal lobe EEG device, Mindeep. (**a**) Hardware circuit; (**b**) shape design and electrode location of the wearable frontal EEG device Mindeep; and (**c**) circuit diagram of Mindeep.

The proposed wearable frontal lobe EEG monitor system, Mindeep, used in this study was developed by our laboratory. The hardware circuit of Mindeep is shown in Figure 1. The low power consumption and low noise integrate an analog front end (AFE) ADS1294 [38] used in Mindeep for 4-channel simultaneous sampling of EEG signals and impedance measurement. The micro control unit (MCU) controls the integrated AFE to collect EEG data. There are three data acquisition modes: (1) transmit data to the computer in the local area network via the ESP32 WIFI module; (2) save data in the SD card; and (3) transmit data to the computer via USB. The data can be saved on the computer in dat format. Mindeep is powered by a lithium battery and the battery life is about 12 h. Data are recorded using a 1000 Hz sampling rate in 24-bit resolution.

According to the EEG 10–10 system, seven Ag/AgCl dry electrodes with 9 mm in diameter (Fp1, Fp2, F7, F8, Fpz, FT7, and FT8) are installed on the fixed position of the headband as shown in Figure 1b. Among the seven electrodes, four electrodes (Fp1, Fp2, F7, and F8) are connected to the analog input of AFE to record EEG signals. The short-circuited FT7 and FT8 electrodes are used as the reference (Ref), and the Fpz electrode is used as the ground (GND). Mindeep supports both dry electrodes and disposable patch Ag/AgCl gel electrodes to collect signals. These dry electrodes can be directly contacted with the skin to measure EEG, or a patch electrode of matching size can be installed on the dry electrodes to measure EEG. Mindeep was worn on the forehead to collect frontal EEG signals.

### 2.2. Simulated Signal Test Task

**Figure 2:**
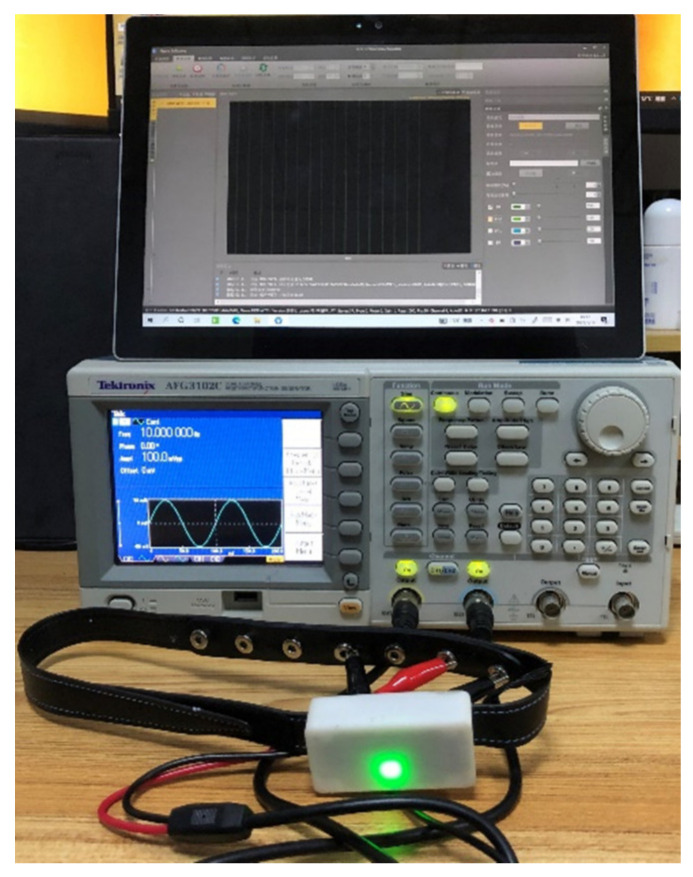
The connection between the arbitrary function generator and Mindeep in the simulated signal test experiment.

In the simulated signal test task, the arbitrary function generator (AFG) (Tektronix, AFG 3102, Beaverton, OR, USA) was applied to simulate sine, square, and triangular waves with different frequencies (10 Hz, 20 Hz, and 30 Hz) and amplitudes (40 mV, 100 mV, and 200 mV). The frequency of the simulated signal was in the frequency range of commonly recorded EEG. Since this AFG cannot generate signals with an amplitude below 1 mV, the amplitude of the simulated signal was higher than that of normal EEG. As shown in Figure 2, the AFG was connected to the dry sensors of Mindeep, the simulated signal was used as the input signal of Mindeep. Mindeep collected signals with 1000 Hz sampling rate.

### 2.3. Signal Quality Comparison Experiments

In this study, 19 healthy students (8 females and 11 males) were recruited from Southeast University (Nanjing, China) to participate in the signal quality comparison experiment. The average age of the participants was 22.79±2.57 years old. All participants were informed about the experimental protocol and matters needing attention, then signed the informed consent before the experiment. This study was approved by IEC for Clinical Research of Zhongda Hospital, affiliated with Southeast University (IRB No. 2019ZDSYLL073-P01).

**Figure 3:**
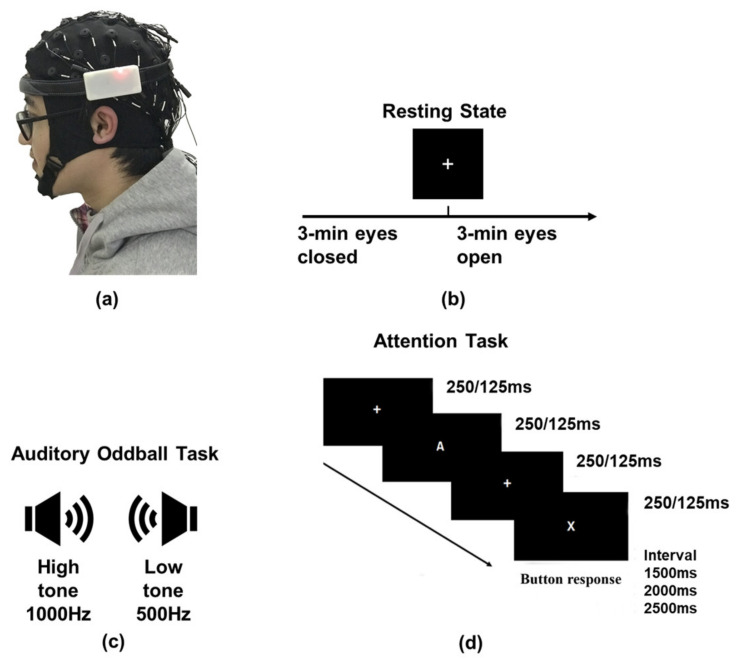
Signal quality comparison experiments. (**a**) Wearing position of Neuroscan and Mindeep; (**b**) resting state task; (**c**) auditory oddball task; and (**d**) two kinds of AX-CPT tasks (AX-CPT-250 ms and AX-CPT-125 ms).

In this study, during the signal quality experiment, EEG signals of four channels (Fp1, Fp2, F7, and F8) were recorded by the standard acquisition device Neuroscan (Synamps2 acquisition system) and the wearable Mindeep device at the same time. Mindeep collected EEG signals through disposable patch Ag/AgCl wet gel electrodes (Ambu, BlueSensor N, Ballerup, Denmark), while Neuroscan collected signals through the 10–10 system electrode cap with conductive paste. To ensure that the EEG collected during the experiment had good signal quality, alcohol and scrub gel were used to clean the forehead to reduce the impedance between the electrodes and the skin before the experiment. Before EEG acquisition, the skin-electrode impedance of both Mindeep and Neuroscan dropped below 10 kΩ. The wearing position of Neuroscan and Mindeep is shown in Figure 3a, after wearing the Neuroscan’s electrode cap as normal, Mindeep was worn below the electrode cap. The EEG collected by Neuroscan used the 10–10 system EEG cap, so the Neuroscan electrode positions defaulted to the standard 10–10 system. By adjusting the wearing position of Mindeep and the angle of the patch electrodes, the electrodes of Mindeep were as close as possible to those of Neuroscan. The head circumference and shape of each subject were not the same, but after adjustment, the distance between Mindeep’s patch electrodes and Neuroscan’s electrodes was within 1 cm. For both devices, the sampling rate of EEG signals was 1000 Hz, and the references were FT7 and FT8.

The signal quality experiment was carried out in a quiet and dimly lit room. All participants were required to sit on the chair with their eyes about 0.5 m away from the screen. The experiment included three parts (see Figure 3b–d): (1) resting task; (2) auditory oddball task [39]; and (3) attention task: AX continuous performance test (AX-CPT) [37,40].

#### 2.3.1. Resting Task

The resting task included 3-min rest with EC and 3-min rest with EO. During the EO resting task, the participants were instructed to remain relaxed and look at a white fixation cross on a black background (see Figure 3b).

#### 2.3.2. Auditory Oddball Task

All participants listened to the 1000 Hz high tone (175 ms duration) and 500 Hz low tone (175 ms duration) presented through a pair of insert earphones (Audio Technica CK330iS) during the auditory oddball experiment (see Figure 3c). The interval between any two adjacent tones was 425 ms. To maintain the participants’ auditory attention, subjects were required to count the number of high tones (deviation tone) occurrences, which randomly interspersed amid a series of low tones (standard tone). The auditory oddball experiment contained 5 sessions; there were 25 high tones and 125 low tones in each session.

#### 2.3.3. AX-CPT Tasks

In this study, the AX-CPT tasks (attention task) were employed to assess the sustained attention capability of the participants (see Figure 3d). During the AX-CPT task, a series of white letters of the English alphabet were presented on the black screen randomly. The participants were instructed to click the left mouse button as fast as possible when the target sequence “X” was preceded by an “A” and to click the right mouse button for other target sequences different from the sequence “AX”. In the AX-CPT-250 ms task, each letter stimulus was presented for 250 ms with an inter-stimulus interval (ISI) of 250 ms and an inter-trial interval (ITI) of 1500, 2000, or 2500 ms randomly set. Inter-stimulus was a white fixation cross on a black background. There were 160 trials, with 120 “AX” sequences and 40 other sequences, each sequence contained two letters. In the AX-CPT-125 ms task, each letter stimulus was presented for 125 ms with an ISI of 125 ms and an ITI of 1500, 2000, or 2500 ms randomly. For each subtask, there were 192 trials, with 144 “AX” sequences and 48 other sequences.

## 3. Methods

### 3.1. EEG Preprocessing

The EEG preprocessing was completed in Matlab R2019; the steps were as follows: (1) The power frequency noise (50 Hz in China) was removed by 50 Hz notch filter; (2) Baseline was removed by 0.3 Hz high pass filter; (3) High-frequency noise (including partial EMG) was removed by 45 Hz low pass filter; (4) Due to the limitation of wearable device’s channel number, eye movement artifacts were removed by discrete wavelet transform (DWT) [41].

### 3.2. Signal-to-Noise Ratio

In this study, the power frequency noise, baseline (below 0.3 Hz), and high-frequency noise (above 45 Hz) were defined as noise N. While SNR represented the power ratio of the clean EEG signal and the noise. The clean EEG signal S was the signal after the raw EEG signal R filtered by the 0.3 Hz high pass filter, 45 Hz low pass filter, and 50 Hz notch filter. The noise N was calculated as:

(1) N=R−S

The SNR was calculated according to the following formula:

(2) $$\mathrm{SNR}=10\times log_{10}\left(\frac{P_{S}}{P_{N}}\right)$$

where $P_{S}$ is the power of clean EEG signal S and $P_{N}$ is the power of noise N.

### 3.3. Valid Signal Proportion

The position and duration of head movement, eye movement, and electromyography (EMG) artifacts of the raw EEG were detected by the following steps:

Step 1: Butterworth bandpass filter was used to extract useful EEG signals.

Step 2: The envelope curve of the artifact was detected by the Hilbert method.

Step 3: Locate the location of artifacts on the absolute envelope curve through the adaptive threshold.

The detailed parameters include the lower and upper cut-off frequency and threshold. The amplitude of the head movement artifact is much higher than EEG, which is 10 times (empirical value) to even dozens of times that of EEG signal, and the frequency range is (0.3, 2) Hz. Therefore, the 0.3–2 Hz Butterworth bandpass filter was applied and the threshold was set to 10×MED, where MED is the median of the absolute value of the envelope. The part that exceeded the threshold was the head movement artifact. The main frequency range of eye movement artifacts is (0.3, 10) Hz. The amplitude of eye movement artifacts is 5 to 10 times (empirical value) that of EEG signals. The 0.3–10 Hz Butterworth bandpass filter was applied and the threshold was set to 5×MED. The place where the amplitude was greater than the threshold was the location of the eye movement artifacts. To avoid repeated recognition of head movement artifacts and eye movement artifacts, the motion artifacts were first identified and eliminated, and then the eye movement artifacts were identified. Different from head movement artifacts and eye movement artifacts, high-frequency EMG is extracted by the 30–200 Hz Butterworth bandpass filter. Since the amplitude of the EMG signal is higher than that of the EEG signal, the threshold was set to MED + 5×STD, where STD is the standard deviation of the absolute value of the envelope and 5 times is the empirical value, and those greater than the threshold were considered as EMG.

The valid signal proportion (VSP) was calculated as:

(3) $$\mathrm{VSP}=L_{valid}/L_{raw}\times 100$$

where $L_{raw}$ was the data length of raw EEG signal, $L_{valid}$ was the data length of EEG signal after clipping the artifact.

### 3.4. Relative Power

The welch PSD [42] is a linear analysis method based on Fourier transform, which is the most commonly used method in conventional EEG analysis. For the single channel EEG oscillations $X=\left\{x_{i}\right\}=\left\{x_{1},x_{2},\ldots ,x_{N}\right\}\;i=1,2,\ldots ,N$, where N is the number of points in the time series. The PSD curve p represented the spectral energy distribution.

The relative PSD curve $\bar{p}$ was calculated as:

(4) $$\bar{p}=p/\sum_{0.3}^{45}p$$

The relative power $\bar{P}$ of [*f*_1_, *f*_2_] frequency band is the ratio of the sum PSD in [*f*_1_, *f*_2_] frequency range to the sum PSD in (0.3 Hz, 45 Hz) frequency range:

(5) $$\bar{P}=\sum_{f_{1}}^{f_{2}}p/\sum_{0.3}^{45}p$$

### 3.5. Mismatch Negativity

MMN [43] is the ERP responses elicited by auditory events that break the expectations created by the regularity of previous standard auditory stimuli. Raw EEG data recorded in the auditory oddball task was bandpass filtered from 1 to 25 Hz. The EEG signal was segmented with limits of −100 ms to 400 ms relative to the stimulus. Baseline correction was then performed on the mean amplitude from −100 ms to 0 ms of each epoch. Data epochs with amplitudes exceeding ±50 μV were discarded. The average standard (ERP) of all data epochs for standard stimulus and the average deviation ERP of all data epochs for deviation stimulus were calculated. MMN responses were obtained by subtracting the standard ERP from the deviation ERP.

### 3.6. Morlet Continuous Wavelet Transform

The Morlet continuous wavelet transform (MCWT) [44] was one of the most commonly used types of wavelet transform methods in the field of physiological signal processing. In this paper, the MCWT with 6 Hz central frequency was applied to describe the time-frequency information of EEG signal. The time resolution is 2 ms, and the frequency resolution is 0.5 Hz.

### 3.7. Sample Entropy

In this study, sample entropy (SE) of five EEG frequency bands (delta: 0.5–4 Hz; theta: 4–8 Hz; alpha: 8–13 Hz; beta: 13–30 Hz; gamma: 30–45 Hz) were extracted as features. The EEG frequency bands were decomposed by DWT with a 7-level and Sym7′ wavelet basis. SE is a modification of approximate entropy [45]. We have an $imf_{N}$ = $x_{i}=\left(x_{1},x_{2},x_{3},\ldots ,x_{N}\right)\;i=1,2,3,\ldots ,N$ and use a time interval to reconstruct series $X_{m}\left(i\right)=\left(x_{i},x_{i+1},x_{i+2},\ldots ,x_{i+m-1}\right)\;i=1,2,3,\ldots ,N-m+1$. The length of the sequence is m. The distance function of two sequences is $d\left[X_{m}\left(i\right),X_{m}\left(j\right)\right]$. For a given embedding dimension m, tolerance *r* and number of data points N, SE is expressed as:

(6) $$\mathrm{SE}\left(r,m,N\right)=-log\frac{A^{m+1}\left(r\right)}{B^{m}\left(r\right)}$$

where, $B^{m}\left(r\right)$ is the number of template vector pairs having $d\left[X_{m}\left(i\right),X_{m}\left(j\right)\right]<r$ and represents the similarity between two sequences of length m, $A^{m+1}\left(r\right)$ is the number of template vector pairs having $d\left[X_{m+1}\left(i\right),X_{m+1}\left(j\right)\right]<r$ and represents the similarity between two sequences of length *m* + 1.

According to our previous studies [31,37], m=2 and r=0.2 were used in this study.

### 3.8. Statistical Analytics

In this paper, the statistical analysis was performed on Matlab R2019. The parametric test one-way analysis of variance (ANOVA) [46] was chosen when the data satisfied the assumptions. The significant difference was defined as the *p*-value < 0.05.

To explore the correlation between EEG indicators and behavioral scale results, the nonparametric Spearman’s rank correlation [47] was used in this study, since the scale scores do not conform to the normal distribution. If the *p*-value of correlation coefficients calculated by t approximation is less than 0.05, then the correlation coefficient is significantly different from zero.

Cross-correlation [48], which measured the similarity of two series relative to time, was used in this paper.

### 3.9. EXtreme Gradient Boosting

EXtreme gradient boosting (XGBoost) [49] is one of the Boosting algorithms whose ideal is to integrate many weak classifiers to construct a strong classifier. Compared to the parallel structure of bagging, boosting works in the serial structure, which means that the structure of each tree depends on the previous trees. XGBoost adds a regularization term to the cost function to control the complexity of the model.

The 10-fold cross-validation was used before XGBoost to divide all samples into 10 parts. One of the 10 parts was used as a testing set and the remaining nine parts were used as a training set. Before training, the features of training and testing sets were normalized by mapping row minimum and maximum values to [−1, 1].

In this study, the parameters of XGBoost are set as follows: learning rate: 0.1; base classifier number: 50; maximum tree depth: 5; subsample ratio of the training set: 0.9; and the L2 regularization term on weights lambda: 2.

## 4. Results

This section included the results of the simulated signal test task and signal quality comparison experiments.

### 4.1. Simulated Signal Test

**Figure 4:**
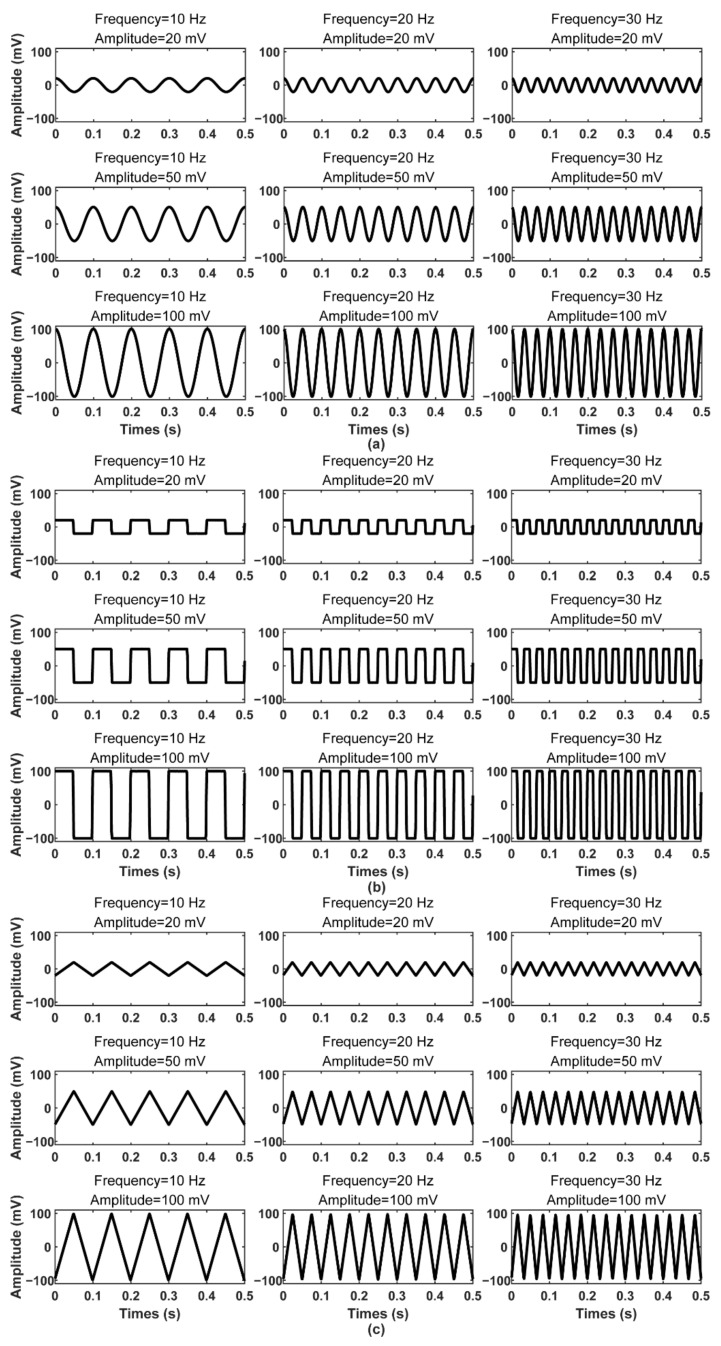
Simulated signals recorded by Mindeep. (**a**) Sine wave; (**b**) square wave; and (**c**) triangular wave with different frequencies (10 Hz, 20 Hz, and 30 Hz) and amplitudes (20 mV, 50 mV, and 100 mV).

The wearable device, Mindeep, recorded the signals simulated by AFG. The recorded signals are shown in Figure 4, including sine wave, square wave, and triangular waves with different frequencies (10 Hz, 20 Hz, and 30 Hz) and different amplitudes (20 mV, 50 mV, and 100 mV). As can be seen, the collected signals had a good degree of restoration and no obvious distortion. The normalized cross-correlation coefficients between the simulated signals and recorded signals are shown in Table 1. The correlation coefficients in different frequencies and amplitudes were all greater than 0.9. The correlation coefficients of sine waves and triangular waves were higher than those of square waves. As the frequency increased, the correlation coefficient decreased slightly, and as the amplitude decreased, the correlation coefficient also decreased slightly.

### 4.2. Resting State Task

#### 4.2.1. Real time EEG signals

**Figure 5:**
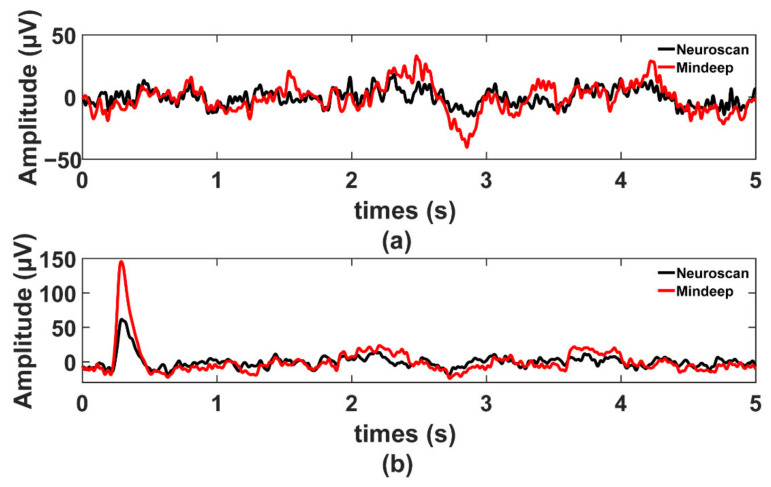
Resting state results. (**a**) EEG signals in EC; and (**b**) EEG signals in EO. The black line is the EEG collected by Neuroscan and the red line is the EEG collected by Mindeep.

The preprocessed EEG signals, filtered by the 0.3 Hz high pass filter, 45 Hz low pass filter, and 50 Hz notch filter, simultaneously recorded by Neuroscan and Mindeep during EC and EO are shown in Figure 5a,b. Compared to Neuroscan, EEG recorded by Mindeep had much more low-frequency components, and the amplitude of eye-movement artifact recorded by Mindeep was higher (which may be caused by the wearing position of Mindeep being closer to the eyes).

#### 4.2.2. SNR, VSR, and Similarity

The SNR and VSR of EEG recorded by the two devices in EC and EO were calculated. As shown in Table 2, the SNRs of Mindeep were slightly lower than Neuroscan. The VSRs of Mindeep were also slightly lower than Neuroscan, but still within an acceptable range. The similarities of the preprocessed EEG signals from the two devices were computed using cross-correlation; the average normalized cross-correlation coefficients (Coef) and the average lags of all participants are shown in Table 2. The EEG similarities in EC were higher than in EO for all channels.

#### 4.2.3. Relative PSD and Berger Effect

**Figure 6:**
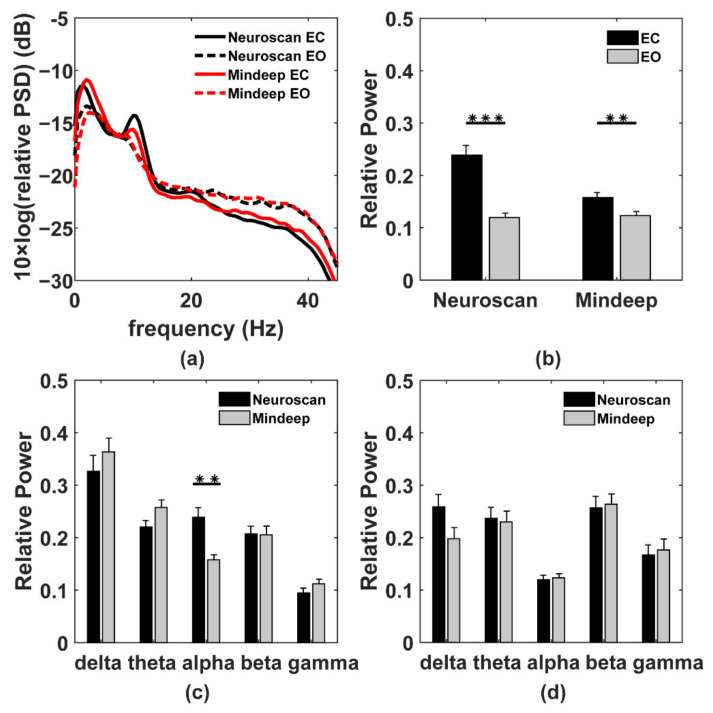
Power spectral density results. (**a**) The relative power spectral density curve for Neuroscan and Mindeep in EC and EO. The black solid line is the PSD curve for Neuroscan in EC. The black dotted line is the PSD curve for Neuroscan in EO. The red solid line is the PSD curve for Mindeep in EC. The red dotted line is the PSD curve for Mindeep in EO. (**b**) The alpha band relative power of Neuroscan and Mindeep in EC (black bar) and EO (grey bar). The relative power of delta, theta, alpha, beta, and gamma band for Neuroscan (black bar) and Mindeep (grey bar) in (**c**) EC state and (**d**) EO state. The significant difference (*** represented *p* < 0.001, ** represented *p* < 0.01) was shown in (**b**–**d**).

The relative PSD curves of the preprocessed EEG in EC and EO are shown in Figure 6a. The four-channel average relative PSD of Mindeep in the whole frequency band (0.3–45 Hz) was similar to Neuroscan in EC and EO. It can be observed that the EO state had higher delta (0.3–4 Hz) and alpha (8–13 Hz) relative PSD, and lower beta (13–30 Hz) and gamma (30–45 Hz) relative PSD. The relative power of delta, theta, alpha, beta, and gamma bands were shown in Figure 6b–d. As we can see in Figure 6b, the relative power of alpha band in EC was higher than EO, confirming that the Berger effect was observed, for both Neuroscan (ANOVA test, *p* < 0.001) and Mindeep (ANOVA test, *p* < 0.01). There was no significant difference in relative power of the five frequency bands (delta, theta, alpha, beta, and gamma) between Neuroscan and Mindeep in the EO state shown in Figure 6d. The alpha relative power of Neuroscan was significantly higher than Mindeep (ANOVA test, *p* < 0.01), but there was no significant difference in other frequency bands (Figure 6c).

### 4.3. Auditory Oddball Paradigm

**Figure 7:**
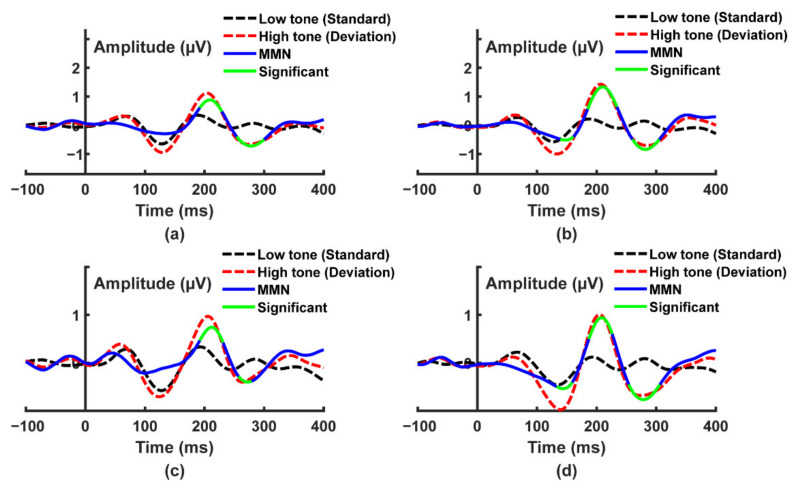
The averaged event related potential (ERP) curves of Mindeep with low tone and high tone stimulation. (**a**) Fp1; (**b**) Fp2; (**c**) F7; and (**d**) F8.

**Figure 8:**
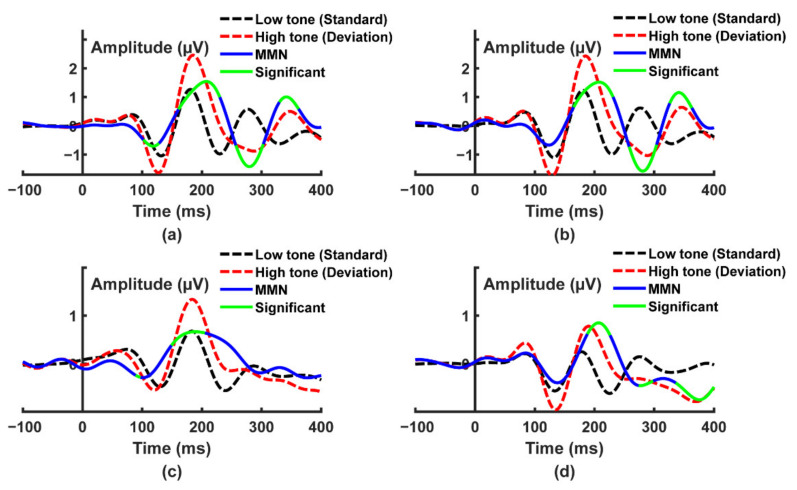
The averaged event related potential (ERP) curves of Neuroscan with low tone and high tone stimulation. (**a**) Fp1; (**b**) Fp2; (**c**) F7; and (**d**) F8.

The averaged ERP curves with low tone (standard) and high tone (deviation) stimulations in the auditory oddball task, which was computed on EEG, are shown in Figure 7 and Figure 8. The black dotted lines were low tone ERP, the red dotted lines were high tone ERP, the blue solid lines were MMN response, and the green lines indicated intervals where the MMN response was statistically significant (ANOVA test, *p* < 0.05). The absolute amplitude of ERP curves recorded by Mindeep was lower than Neuroscan for low tone and high tone. The absolute amplitude of high tone ERP curves was higher than low tone for both Neuroscan and Mindeep. The P1, N1, P2, and N2 can be observed in four channels for both Neuroscan and Mindeep. As shown in Figure 7, for wearable device Mindeep, the statistical differences of MMN response mainly appeared at P2 and N2, and the MMN of Fp2 and F8 also had statistical differences at P1. As shown in Figure 8, for gold-standard Neuroscan, the statistical differences of MMN response in Fp1 were at N1, P2, N2, and P3; in Fp2, at P2, N2, and P3; in F7, at P2; in F8, at P2 and N2.

### 4.4. AX-CPT Attention Task

**Figure 9:**
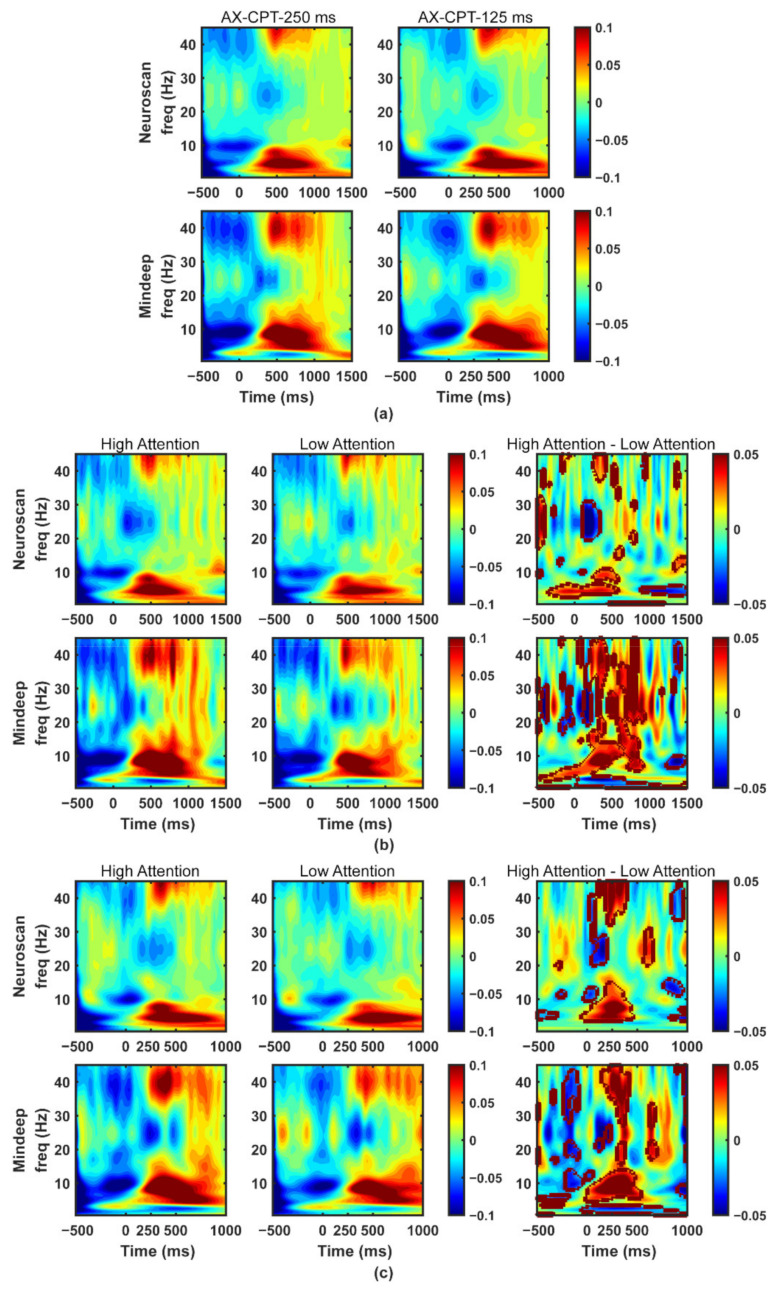
Time-frequency results of AX-CPT tasks. (**a**) Time-frequency analysis graphs of EEG simultaneously recorded by Neuroscan and Mindeep during attention task 1 and task 2. Time-frequency graphs for high attention and low attention, and the time-frequency differences between high attention and low attention for Neuroscan and Mindeep, respectively, during attention tasks (**b**) AX-CPT-250 ms and (**c**) AX-CPT-125 ms.

The time-frequency results of the preprocessed EEG during the stimulation period of AX-CPT-250 ms (1000 ms) task and AX-CPT-125 ms (500 ms) task were shown in Figure 9a. For both Mindeep and Neuroscan, during AX-CPT-250 ms task, the power of theta (4–8 Hz) and gamma (30–45 Hz) started to increase, and the power of beta (13–30 Hz) started to gradually decrease at 250 ms; consistently, during AX-CPT-125 ms task, the start point was observed at 125 ms.

The distributions of reaction time of all participants during AX-CPT-250 ms and AX-CPT-125 ms tasks fitted the normal distribution. When people remain highly focused, they usually respond immediately and rapidly to visual stimuli. Conversely, people with low attention levels usually have slower responses. Therefore, the high-attention group consists of the EEG epochs with reaction time (RT) in the top 20% in all experiment epochs, and the low-attention group consists of the EEG epochs with RT in the bottom 20%. As shown in Figure 9b,c, the area within the solid black line indicated statistical differences between the low-attention group and the high-attention group (ANOVA test, *p* < 0.05). For those two attention AX-CPT tasks, the differences of time-frequency graphs between high-attention group and low-attention group mainly appeared in the stimulation period. The differences between the low-attention group and the high-attention group in Mindeep’s time-frequency results were larger than that of Neuroscan. Compared with the inter-trial interval (without stimulus), the high-attention group evoked higher theta, alpha, and gamma power, and lower beta power during stimulation.

**Figure 10:**
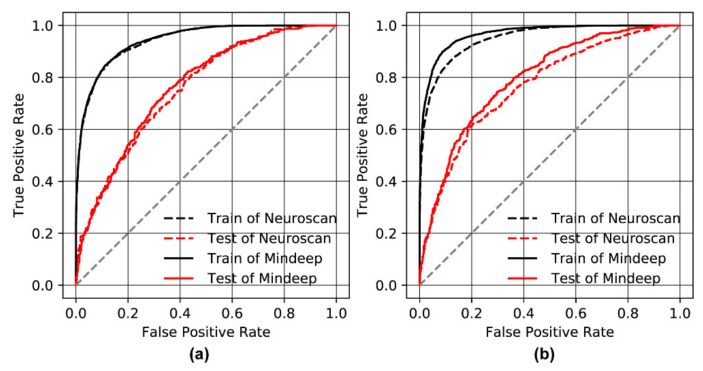
Accuracies and ROC curves of XGBoost model. (**a**) AX-CPT-250 ms; and (**b**) AX-CPT-125 ms.

The stimulus duration of each trial was 1000 ms in the AX-CPT-250 ms task and 500 ms in the AX-CPT-125 ms task. According to the results of the time-frequency analysis shown in Figure 9, the relative power and SE features of the five frequency bands during the stimulation period of each trial were extracted. The classification results and ROC curve of the XGBoost model are shown in Table 3 and Figure 10. The classification accuracy in distinguishing between high attention and low attention of Mindeep (AX-CPT-250 ms: accuracy of 71.12%; X-CPT-125 ms: accuracy of 74.76%) was slightly higher than that of Neuroscan (AX-CPT-250 ms: accuracy of 70.19%; AX-CPT-125 ms: accuracy of 72.80%), and the classification accuracy of the AX-CPT-125 ms task was higher than that of the AX-CPT-250 ms task.

## 5. Discussion

In the past few decades, the emergence of novel signal acquisition technologies has contributed to advancing study on human brain research and application. A new generation of portable EEG devices allows research in new experimental settings. This study mainly proposed a new wearable EEG device, Mindeep, and conducted device verification experiments and practice-oriented research on it. In more detail, we aimed to assess (1) if the Mindeep circuit can perfectly collect various simulated signals, (2) the performance of the time and frequency domain indicators of the signals collected by Mindeep, (3) if Mindeep can record reliable auditory evoked potential responses, and (4) if Mindeep can be applied to monitor and distinguish the attention status of healthy subjects. Results demonstrated that Mindeep can record high-quality EEG signals comparable to those obtained using the gold standard Neuroscan.

In this study, Mindeep was worn under the Neuroscan (see Figure 3a), which means Mindeep’s electrodes were closer to the eyes and farther from the occipital lobe than Neuroscan. Therefore, Mindeep had a larger low-frequency proportion, lower SNR, lower VSP, and lower alpha band relative power. The MMN response of Mindeep in F7 and F8 showed the greater differences between high tone and low tone than Neuroscan. The results of AX-CPT task showed that Mindeep can be used to recognize high attention and low attention. The attention recognition results of Mindeep were better than Neuroscan (see Figure 9). It can be concluded that the newly proposed wearable device Mindeep can collect EEG signals with good signal quality and can be used to distinguish different levels of attention. The attention recognition accuracy of Neuroscan was slightly lower than that of Mindeep, which may be caused by the fact that the high-frequency part of Mindeep contained more EMG signals (wearing position is close to the eye).

As shown in Table 4, compared with other portable EEG devices (Emotiv, Muse, Neurosky [50], and B-Alert), Mindeep opens up raw data to the public. The data collected by Mindeep is transmitted wirelessly via Wi-Fi to realize the real-time transmission of high sampling rate EEG data (adjustable, 250/500/1000 Hz). Mindeep’s battery life is up to 12 h, which can fully satisfy the sleep EEG monitoring for research. To better meet the needs of scientific research and clinical practice, Mindeep provides event-mark and multi-device synchronization functions. The impedance response function was added to help users accurately know whether the current impedance falls within the required range.

EEG electrodes can be divided into three types: wet electrodes, dry electrodes, and semi-dry electrodes [51]. The skin-electrode impedance of the wet electrodes is usually the lowest compared with other electrodes, but problems such as increased impedance and signal attenuation may occur with prolonged use due to the volatilization of the gel liquid. Traditional EEG caps require manual injection of conductive paste, which is complicated to operate and is not suitable for non-laboratory environments. However, the disposable patch-type wet gel electrodes, which preplace a small amount of gel in the electrodes, are easy to use and suitable for daily wearing [52]. Dry electrodes are easy to use and reusable, suitable for wearable EEG devices in non-clinical applications and long-term monitoring, but the EEG signals recorded by dry electrodes can be more likely interfered by movement artifacts. The skin-electrode impedance of traditional dry electrodes is usually high. In recent years, dry electrodes made of new materials have been proven to effectively reduce the impedance of skin electrodes [53,54]. Semi-dry electrodes are a combination of wet and dry electrodes, retaining the advantages of wet and dry electrodes, but they are still in the early research stage [55,56]. Considering the advantages and disadvantages of the three electrodes, the wearable EEG device Mindeep proposed in this study supports two sensing methods: traditional AgCl dry electrodes and patch-type Ag/AgCl wet gel electrodes. In general, it is recommended to use patch electrodes, but if the acquisition time is long or the cost of patch electrodes is considered too high, AgCl dry electrodes can be used instead. Mindeep only needs 2–5 min to complete wearing the device with dry electrodes or path-type electrodes, while Neuroscan requires at least 30 min preparation time, including scalp cleaning before and after use, wearing an EEG electrode cap, and injecting conductive paste. These advantages of Mindeep promote the improvement of user acceptance and the long-term collection of EEG signals in daily life.

There were still some limitations to this study. EEG signals are physiological signals lower than 20 µV, but AFG used in this study can only simulate signals higher than 20 mV. The results in Section 4.1 showed that as the amplitude decreased, the correlation coefficient between the simulated signals and the recorded signals gradually decreased. If the amplitude of the simulated signal is lower than 20 µV, the correlation coefficient may be lower than 0.9. In future research, we will use the µV-level AFG to simulate the signal below 1 mV to test the quality of the collected signals of Mindeep.

Although attention recognition is not the main purpose of this study, the accuracies of distinguishing between high and low attention in this study are 71.12% and 74.76%, which is lower than previous studies [37]. The reasons may be: (1) the duration of AX-CPT was only about 6 min, and the subjects were healthy college students, and their attention may always be kept at a high level during the AX-CPT task; (2) only the four prefrontal lobe channels were collected in this study, and the number of channels was small; and (3) the distance between the reference electrode and the EEG electrodes were too close. In follow-up studies, we will further improve the hardware design and electrode placement of Mindeep and validate the value of Mindeep in other research.

## 6. Conclusions

In this study, a novel wearable frontal lobe EEG monitoring device Mindeep was proposed, and a rigorous signal quality evaluation analysis was performed on Mindeep. Additionally, we further verify the feasibility of Mindeep in practical applications on tracking the attention state. The results of the simulated signal test task proved that Mindeep’s hardware circuit works normally, and the collected sine, square, and triangular signals with different frequencies and amplitudes were not distorted. Another experiment comparing the signal quality of Mindeep with the gold standard EEG acquisition device Neuroscan, confirmed that Mindeep can reproduce the results of Neuroscan. Based on resting state EEG data, the reliable signal quality of Mindeep was proven through the SNR, VSP, and similarity indexes. In the auditory oddball experiment (see Figure 7 and Figure 8), the well-known events N1, P1, N2, and P2 for high tone and low tone were observed for both Mindeep and Neuroscan. The MMN response, which was used to distinguish high tone (deviation) and low tone (standard) of Mindeep, had significant differences at N2 and P2. Through the time-frequency diagram (see Figure 9) and classification results (see Figure 10) of Mindeep and Neuroscan in the AX-CPT tasks, the low attention epochs can be recognized from high attention epochs by Mindeep. These results indicate that this wearable frontal lobe EEG monitoring device can reliably record event-evoked potentials and differences between target and non-target stimuli, which can be used for data acquisition and practical applications of various brain computer interfaces. The above results also fully prove that Mindeep can be used in scientific research, medical treatment, and daily life.

## Figures and Tables

**Table 1:** Normalized cross-correlation coefficients for simulated input signals and recorded output signals.

Simulated Wave		Frequency (Hz)	10	20	30
	Amplitude (mV)				
Sine	20		0.999	0.994	0.988
	50		0.999	0.994	0.993
	100		0.999	0.997	0.993
Square	20		0.988	0.969	0.944
	50		0.991	0.973	0.958
	100		0.988	0.975	0.957
Triangular	20		0.999	0.995	0.985
	50		0.999	0.995	0.987
	100		0.999	0.995	0.990

**Table 2:** SNR, VSP, and normalized cross-correlation coefficients for Neuroscan and Mindeep on all channels in EC and EO.

State	Channel	Device	SNR (dB)	VSP (%)	Coef	Lag (ms)
EC	Fp1	Neuroscan	−49.80 ± 2.08	91.78 ± 1.43	0.73 ± 0.02	1.18 ± 0.15
		Mindeep	−54.27 ± 2.24	87.30 ± 2.01		
	Fp2	Neuroscan	−46.68 ± 2.84	91.53 ± 1.45	0.73 ± 0.02	1.14 ± 0.14
		Mindeep	−54.51 ± 2.21	87.10 ± 1.70		
	F7	Neuroscan	−48.16 ± 2.16	90.96 ± 1.59	0.75 ± 0.02	1.12 ± 0.14
		Mindeep	−54.82 ± 1.54	86.62 ± 1.99		
	F8	Neuroscan	−45.75 ± 2.40	91.93 ± 1.35	0.75 ± 0.02	1.02 ± 0.12
		Mindeep	−53.16 ± 2.05	85.28 ± 2.08		
EO	Fp1	Neuroscan	−44.54 ± 1.95	75.34 ± 2.69	0.71 ± 0.02	0.88 ± 0.13
		Mindeep	−49.27 ± 2.01	66.74 ± 2.93		
	Fp2	Neuroscan	−41.54 ± 2.73	74.68 ± 2.33	0.71 ± 0.02	0.79 ± 0.11
		Mindeep	−49.77 ± 1.84	67.95 ± 2.81		
	F7	Neuroscan	−44.14 ± 2.21	84.39 ± 1.52	0.71 ± 0.02	0.97 ± 0.12
		Mindeep	−50.16 ± 1.63	68.82 ± 3.31		
	F8	Neuroscan	−42.23 ± 2.33	83.90 ± 1.66	0.71 ± 0.02	1.18 ± 0.10
		Mindeep	−47.87 ± 2.01	67.65 ± 2.71		

**Table 3:** Comparison of attention classification models for Neuroscan and Mindeep in AX-CPT-250 ms and AX-CPT-125 ms task.

		Precision (%)	Recall (%)	F1 (%)	Accuracy (%)
AX-CPT-250 ms	Neuroscan	69.95	69.65	69.80	70.19
	Mindeep	72.26	67.56	69.83	71.12
AX-CPT-125 ms	Neuroscan	73.99	68.26	71.01	72.80
	Mindeep	77.23	68.48	72.59	74.76

**Table 4:** Comparison of portable EEG devices.

	Emotiv				Muse		Neurosky	B-Alert		Mindeep
Model	MN8	Insight	Epoc + /Epoc x	Epoc Flex Kit	Muse 2	Muse S	Mindwave	B-Alert X10	B-Alert X24	MDP4CH-WTT
Price (dollar)	--	299	849	1699 (Saline)/2099 (Conductive paste)	249.99	349.99	109.99	--		799
Cost (dollar)	×	29/99/199/month			×	×	×	×		×
Sensor Count	2	5	14	32	4		1	9	20	4
Sensor Location	Left and Right Ear	AF3, AF4, T7, T8, Pz	AF3, AF4, F3, F4, FC5, FC6, F7, F8, T7, T8, P7, P8, O1, O2	10–20 location	Fp1, Fp2, TP9, TP10		Fpz	POz, Fz, Cz, F3, F4, C3, C4, P3, P4	10–20 location	Fp1, Fp2, F7, F8
Reference	A1/A2	PZ	P3/P4	FCz	Fpz		A1	A1/A2		Fpz
Sample rate (Hz)	128	128	128/256	128	220/500		512	256		250/500/1000
Frequency Response (Hz)	0.16–45	0.5–43	0.16–43		0.1–30		3–100	0.1–67		raw
Resolution (bits)	14				10		12	16		24
Electrode	Dry		Saline	Saline/Conductive paste	Dry (silver)	Dry (gold)	Dry	Conductive paste		Dry/patch electrodes
Wireless	Bluetooth				Bluetooth		Bluetooth	Bluetooth		Wi-Fi
Battery life (hours)	6	4	6 (Epoc +)/6 (Epoc x)	9	5	10	8	8		12
Acceleration	6-axis	9-axis	9-axis	9-axis	9-axis		×	3-axis		9-axis
Multidevice synchronization	×	×	×	×	×		×	×		√
Event marker	×	×	×	×	×		×	√		√

## Data Availability

The EEG datasets generated for this study are available on request to the corresponding author.
